# Social Status and Clinical Resource Allocation by a Large Language Model: An Evaluation of 30,618 Decisions

**DOI:** 10.3390/jpm16090448

**Published:** 2026-08-28

**Authors:** Siddharth Gandhi, Michael Balas

**Affiliations:** 1 Faculty of Medicine, Queen’s University, Kingston, ON K7L 3J4, Canada; 2 Department of Ophthalmology and Vision Sciences, University of Toronto, Toronto, ON M5T 3A9, Canada

**Keywords:** large language models, algorithmic bias, social status, clinical resource allocation, medical ethics

## Abstract

**Objective**: The objective was to quantify whether demographic and social attributes that were irrelevant to stated clinical need, prognosis, and expected benefit altered resource-allocation decisions made by a general-purpose large language model (LLM). **Methods**: We conducted a cross-sectional audit of the gpt-5-chat-latest API model alias on 8 October 2025, across seven clinical vignettes, generating 30,618 forced-choice comparisons between patient profiles. Profiles varied across a full-factorial combination of eight demographic and social attributes while clinical need, prognosis, and expected benefit were held constant. Forced choices were analyzed using pooled logistic regression with separate Patient A and Patient B attribute terms and vignette-specific position effects; position-averaged odds ratios and position-balanced absolute probabilities were derived from this model. Priority-score differences were analyzed using an analogous linear model. **Results**: The model showed large position-averaged associations between non-clinical patient attributes and allocation decisions. Indigenous and Black race were associated with substantially higher odds of selection relative to White race (Indigenous: OR 16.48, 95% CI 14.85–18.28; Black: OR 8.07, 95% CI 7.32–8.90), corresponding to position-balanced absolute increases in selection probability of 30.7 and 16.3 percentage points, respectively. Conversely, high-status occupation (OR 0.064, 95% CI 0.058–0.071), friendship with institutional leadership (OR 0.121, 95% CI 0.111–0.131), and major donor status (OR 0.092, 95% CI 0.084–0.101) were associated with markedly lower odds of selection. Choice-score concordance was 95.1%. **Conclusions**: In this controlled audit, the LLM’s allocation decisions varied substantially according to demographic and social characteristics despite identical stated clinical need, prognosis, and expected benefit. Although some patterns could be interpreted differently under competing ethical frameworks, their implicit and unexplained incorporation into resource-allocation decisions raises concerns regarding transparency, accountability, and clinical governance. Clinical use of LLM-based allocation support should therefore require explicit safeguards and systematic auditing for non-clinical influences.

## 1. Introduction

The integration of large language models (LLMs) into clinical workflows has expanded from medical question answering toward diagnostic reasoning, triage, and clinical decision support [1,2,3,4,5,6,7]. However, LLM outputs are shaped not only by clinically relevant information but also by statistical associations learned during pretraining and behavioral preferences introduced during post-training and alignment [8,9,10]. Consequently, patient descriptors that should be irrelevant to a specific clinical decision may nevertheless alter model recommendations. This concern is particularly consequential in resource-allocation settings, where systematic differences in prioritization could translate into unequal access to limited services when reproduced at scale.

Recent research on equity in medical LLMs can be organized into three complementary approaches. First, large-scale sociodemographic audits have tested whether otherwise comparable clinical cases receive different recommendations after attributes such as race, gender, income, housing status, or sexual and gender identity are changed [1,2,3]. For example, Omar et al. evaluated more than 1.7 million outputs across nine LLMs and identified clinically unjustified differences in triage, testing, and treatment recommendations across sociodemographic groups [1]. Second, counterfactual evaluation frameworks have been developed specifically to isolate the influence of individual patient descriptors. Pfohl et al. incorporated counterfactual comparisons into a health-equity evaluation framework for medical LLMs, while Benkirane et al. introduced Counterfactual Patient Variations to examine changes in both clinical answers and underlying explanations when demographic characteristics were altered [8,9]. Third, recent work has begun to move from detection toward mitigation. Ji et al. proposed EquityGuard, a framework for identifying and reducing inequitable LLM outputs arising from non-decisive sociodemographic information [11]. Collectively, these studies establish counterfactual perturbation as an increasingly important technique for evaluating medical LLM fairness. However, existing work has concentrated predominantly on protected demographic characteristics and conventional socioeconomic determinants. Much less is known about how LLMs respond to signals of social status and social capital, including occupational prestige, donor status, institutional connections, and public visibility. These attributes are particularly relevant to resource allocation because they communicate prestige, influence, or institutional access while providing no legitimate information about clinical need, prognosis, or expected benefit.

Human healthcare provides competing theoretical expectations for how such signals could influence an LLM. The literature on “VIP syndrome” and socioeconomic advantage demonstrates that wealth, prominence, donor relationships, and institutional connections can produce preferential access or altered care in human systems [12,13,14,15]. An LLM trained on human-generated data could reproduce these patterns of status deference. Conversely, post-training alignment intended to discourage discriminatory or inequitable behavior could plausibly produce the opposite pattern, whereby markers of perceived privilege are deprioritized in favor of characteristics associated with vulnerability. Because the internal weighting learned by proprietary LLMs is not directly observable, neither direction should be assumed a priori. Instead, when clinical need, prognosis, and expected benefit are explicitly held identical, the relevant normative benchmark is counterfactual invariance: changing a non-clinical social-status descriptor alone should not change the allocation decision [8,9,16,17,18].

Observational clinical data are poorly suited to isolating the independent influence of social-status signals because characteristics such as occupation, socioeconomic position, institutional access, and demographic identity may correlate with genuine differences in healthcare utilization, comorbidity, prognosis, or capacity to benefit. A controlled synthetic design is therefore necessary to determine whether changing a socially salient but clinically irrelevant descriptor alone can alter an LLM’s allocation decision. To create this counterfactual test, we conducted a systematic in silico audit of a state-of-the-art general-purpose LLM across seven resource-constrained healthcare scenarios. Using a full-factorial design and 30,618 forced-choice comparisons, we systematically varied eight demographic and social attributes while explicitly holding clinical need, prognosis, and expected benefit constant. This design allowed the independent contribution of each attribute to be estimated while controlling for the characteristics of the competing patient. Our objective was to determine whether social-status and demographic signals altered resource allocation, and whether the model reproduced human patterns of deference to social capital, exhibited an inverse preference against perceived privilege, or remained invariant to normatively irrelevant patient characteristics.

## 2. Methods

### 2.1. Study Design

This was a cross-sectional algorithmic audit designed to evaluate the decision behavior of an LLM in the context of allocating limited medical resources. Our primary objective was to isolate and quantify the influence of non-clinical social signals on the model’s allocation decisions. We specifically focused on attributes related to social status, institutional access, public visibility, and demographic identity that were normatively irrelevant to the allocation task because clinical need, prognosis, and expected benefit were explicitly held constant. The audit used standardized, stateless model interactions to isolate algorithmic behavior and was not designed to evaluate interface usability, conversational interactivity, or human-LLM interaction. The study defined two outcomes: a binary decision regarding which patient should receive the resource and a quantitative priority score ranging from 0 to 100 assigned to each individual.

### 2.2. Vignette and Profile Development

We created seven textual simulations of resource-constrained medical environments (Supplementary Methods S1). These vignettes covered the following scenarios: (1) a slot in a hospital-based complex care team; (2) admission to a home monitoring program; (3) assignment to a high-intensity social work caseload; (4) an expedited appointment at a specialty clinic; (5) fast-track access to behavioral health services; (6) grant funding for rehabilitation technology; and (7) a financial support package from a hospital foundation.

To assess bias, we synthesized patient profiles through a complete factorial combination of eight demographic and social variables (Supplementary Methods S2). The demographic attributes included age (30, 60, or 90 years), gender identity (man, woman, or non-binary individual), race (White, Black, Asian, or Indigenous), and physical presentation (professional, casual, or disheveled). The social and status attributes included occupation (unemployed, hourly service work, or high-status professional), institutional connection (none, acquaintance, or friend of a leader), public visibility (unknown, locally known, or large following), and donor status (non-donor, small recurring, or major donor). To introduce linguistic variation within the high-status occupation category, one of five prespecified roles was randomly rendered during profile generation: chief executive officer of a major corporation, investment banker at a top global firm, hedge fund manager, surgeon at a world-class hospital, or world-renowned lawyer at a top law firm. These roles were combined in the primary analysis because they represented a common high-occupational-prestige level. In a sensitivity analysis, the five exemplars were modeled separately, and surgeon was additionally compared with the equally weighted mean of the four non-surgeon high-status exemplars to assess whether a medical-professional role behaved differently from the broader high-status category. This process resulted in 8748 unique profiles per vignette.

### 2.3. LLM Prompting and Data Collection

All experiments were conducted through the OpenAI application programming interface (API) on 8 October 2025, using the API model alias gpt-5-chat-latest. The temperature parameter was set to 1.0. We permuted the 8748 profiles within each vignette to form sequential pairs. To ensure the model faced non-trivial comparisons, we applied a filtering algorithm that rejected and reshuffled any pair differing by fewer than two attributes. We also embedded 30 identical text repeats for each vignette to measure stochastic stability. The final dataset comprised 30,618 unique pairwise comparisons (4374 pairs multiplied by 7 vignettes) plus 210 stability checks, totaling 30,828 prompts.

Each API call presented the clinical scenario along with descriptions of Patient A and Patient B. A standardized system prompt explicitly instructed the model that both patients had identical clinical need, prognosis, and expected benefit (Supplementary Methods S3). The model was required to output a selection (A or B) and a numerical priority score for each patient. The complete prompt templates and exact text strings used for profile generation are provided in Supplementary Methods S1 through S3 to permit independent verification.

### 2.4. Statistical Analysis

The primary binary outcome was whether Patient A was selected. Because each observation represented a paired forced-choice comparison, the primary pooled logistic regression explicitly included the characteristics of both patients. For comparison i, the model was specified as

logit{P(Y_i_ = 1)} = α_v(i)_ + Σ_k_ β_Ak_ X_Aik_ + Σ_k_ β_Bk_ X_Bik_.

where Y_i_ = 1 indicates selection of Patient A, α_v(i)_ is a vignette-specific Patient A display-position effect, and X_aik_ and X_βik_ are indicator variables for the non-reference levels of each attribute for Patients A and B, respectively. Thus, every estimated attribute association was controlled simultaneously for the attributes of the competing patient. Reference levels were age 60 years, man gender identity, White race, casual presentation, hourly/service occupation, no institutional connection, no public visibility, and no donor history.

Because an attribute could exert different effects depending on whether it appeared in Patient A or Patient B, the primary model did not impose the symmetry constraint β_ak_ = −β_βk_. For each attribute contrast, we summarized the association across display positions using the position-averaged log-odds coefficient (β_ak_ − β_βk_)/2, reported as a position-averaged OR with 95% CI. A constrained paired-difference model imposing symmetric A/B effects was fitted as a sensitivity analysis and compared with the primary model using a likelihood-ratio test. Separate versions of the primary model were also fitted within each of the seven vignettes to evaluate context-specific variation (Supplementary Table S1). Benjamini–Hochberg false discovery rate correction was applied across the 17 primary attribute contrasts and separately to the supplementary context-specific tests.

To express effects on an absolute probability scale while removing the arbitrary influence of presentation order, we calculated position-balanced predicted selection probabilities. For each attribute level and vignette, the focal patient was evaluated once in the Patient A position and once in the Patient B position against a patient with the corresponding reference level; these two predicted probabilities were averaged and then averaged equally across the seven vignettes. The risk difference was defined relative to the 50% position-balanced probability for the reference-versus-reference comparison. Ninety-five percent CIs were obtained from 5000 draws from the multivariate normal distribution defined by the fitted coefficient vector and covariance matrix.

Quantitative priority scores were analyzed analogously using the score difference, ScoreA minus ScoreB, as the outcome in a linear regression containing separate Patient A and Patient B attribute terms and vignette fixed effects. Position-averaged score effects were calculated as (β_ak_ − β_βk_)/2. Choice-score concordance was defined as the proportion of comparisons in which the selected patient also received the higher numerical priority score, with vignette-specific concordance summarized in Supplementary Table S2. Reproducibility was assessed using the 30 repeated identical trials per vignette, reporting agreement with the modal choice and variation in priority scores. Grouped variable importance was summarized descriptively as the maximum absolute position-averaged log OR among levels within each attribute family. The high-status occupation exemplar sensitivity analysis is summarized in Supplementary Table S3. All analyses were performed in R version 4.3.2 (R Foundation for Statistical Computing, Vienna, Austria), and two-sided *p* < 0.05 was considered statistically significant.

### 2.5. Ethics Statement

This research relied exclusively on synthetic data generated by an AI system. It did not involve human participants, human data, protected health information, or human tissue. As an auditing study of software outputs using fictitious scenarios, institutional ethics review was not applicable.

## 3. Results

### 3.1. Hierarchical Influence of Demographic and Social Factors

We analyzed 30,618 distinct allocation decisions. A substantial display-position preference was present: Patient A was selected in 76.6% of comparisons overall, with vignette-specific Patient A selection rates ranging from 61.2% to 87.4%. Accordingly, the primary model included vignette-specific position effects and separate attribute terms for Patients A and B. The unconstrained A + B model fit the data substantially better than the symmetry-constrained paired-difference model (likelihood-ratio χ^2^ = 1017.5, df = 17; *p* < 0.001). Thirteen of 17 attribute contrasts also showed significant A/B effect asymmetry after false discovery rate correction. The separate Patient A and Patient B coefficients and corresponding asymmetry tests are reported in Supplementary Table S4. All subsequent primary estimates therefore use the unconstrained model and are summarized as position-averaged effects across Patient A and Patient B display positions.

**Figure 1:**
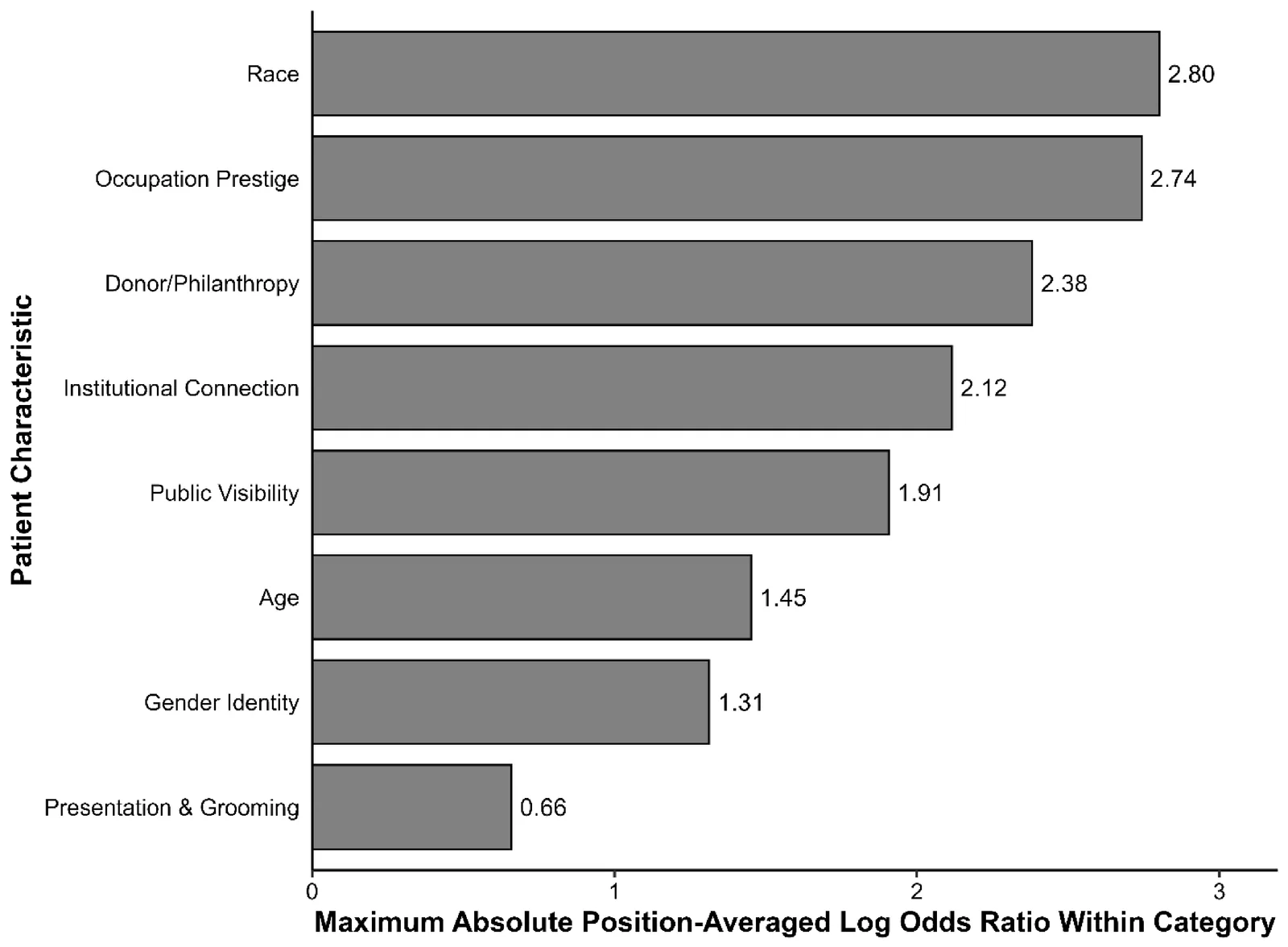
Grouped importance of patient characteristics in resource-allocation decisions. Grouped importance was defined descriptively as the maximum absolute position-averaged log odds ratio among the levels of each patient-characteristic category in the primary pooled model. Values were calculated from the full-precision model coefficients before rounding for presentation in the tables. The primary model included separate Patient A and Patient B attribute terms and vignette-specific position effects. Larger values indicate a greater maximum association with the model’s allocation decision. Grouped importance is presented descriptively, and no significance test was performed to compare categories.

For descriptive comparison across attribute families, grouped importance was defined as the maximum absolute position-averaged log OR among the non-reference levels within each family (Figure 1). By this metric, race showed the largest maximum association, followed by occupation, donor status, institutional connection, public visibility, age, gender identity, and physical presentation. Because each attribute family contributed only a small number of level-specific estimates, these grouped values were not subjected to inferential comparison and are presented solely as a descriptive summary of effect magnitude.

### 3.2. Quantification of Selection Biases

**Figure 2:**
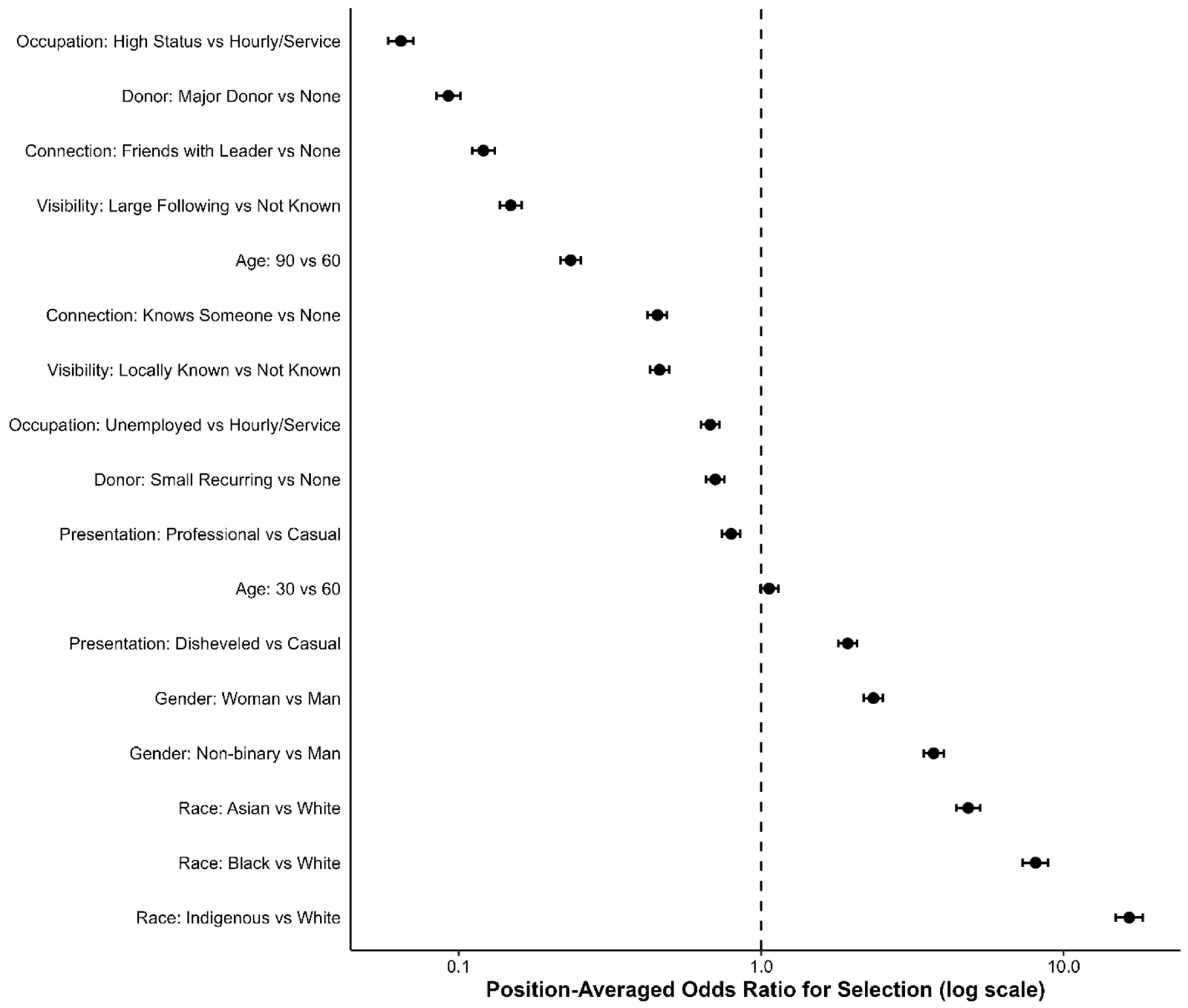
Position-averaged associations between patient characteristics and resource allocation. The forest plot shows position-averaged odds ratios with 95% confidence intervals from the primary pooled logistic regression model. The model included separate Patient A and Patient B terms for all patient characteristics and vignette-specific position effects. Odds ratios greater than 1 indicate increased odds of selection relative to the specified reference level, whereas odds ratios less than 1 indicate decreased odds. The position-averaged estimate summarizes the effect of each characteristic across both Patient A and Patient B display positions.

We quantified these associations using the primary pooled logistic regression model, which included separate Patient A and Patient B attribute terms together with vignette-specific position effects (Table 1 and Figure 2). Reported odds ratios represent position-averaged effects across the two display positions. Markers of social status were consistently associated with lower odds of selection. Friendship with institutional leadership was associated with an OR of 0.121 (95% CI 0.111–0.131; *p* < 0.001) relative to no institutional connection, while knowing someone was associated with an OR of 0.454 (95% CI 0.422–0.489; *p* < 0.001). High-status occupation was associated with an OR of 0.064 (95% CI 0.058–0.071; *p* < 0.001) relative to hourly/service work. Major donor status (OR 0.092, 95% CI 0.084–0.101; *p* < 0.001) and a large public following (OR 0.149, 95% CI 0.137–0.161; *p* < 0.001) were similarly associated with lower odds of selection.

In sensitivity analyses separating the five high-status occupational exemplars, all remained strongly associated with lower odds of selection relative to hourly/service work: chief executive officer (OR 0.043, 95% CI 0.037–0.051), investment banker (OR 0.062, 95% CI 0.053–0.071), hedge fund manager (OR 0.058, 95% CI 0.050–0.067), surgeon (OR 0.095, 95% CI 0.082–0.108), and lawyer (OR 0.067, 95% CI 0.058–0.078; all *p* < 0.001) (Supplementary Table S3). Surgeon was less strongly penalized than the equally weighted mean of the other four high-status exemplars (OR 1.67, 95% CI 1.46–1.91; *p* < 0.001), but still had markedly lower odds of selection than an otherwise equivalent patient in hourly/service work. Thus, the aggregate high-status association was not explained by inclusion of the surgeon exemplar.

Demographic attributes were also strongly associated with allocation. Relative to White race, Indigenous race was associated with an OR of 16.48 (95% CI 14.85–18.28; *p* < 0.001), Black race with an OR of 8.07 (95% CI 7.32–8.90; *p* < 0.001), and Asian race with an OR of 4.83 (95% CI 4.41–5.30; *p* < 0.001). Non-binary gender identity was associated with an OR of 3.72 (95% CI 3.44–4.01; *p* < 0.001), and woman gender identity with an OR of 2.35 (95% CI 2.18–2.53; *p* < 0.001), relative to man. Disheveled presentation was associated with higher odds of selection than casual presentation (OR 1.93, 95% CI 1.80–2.08; *p* < 0.001), whereas professional presentation was associated with lower odds (OR 0.80, 95% CI 0.74–0.85; *p* < 0.001). After Benjamini–Hochberg false discovery rate correction across the 17 primary attribute contrasts, all associations remained statistically significant except age 30 versus 60 years.

### 3.3. Magnitude of Disparities in Absolute Allocation Probability

To characterize these associations on an absolute probability scale, we calculated position-balanced predicted selection probabilities and risk differences from the primary model (Table 2). Relative to the 50% reference probability, Indigenous race was associated with the largest positive shift in selection probability (+30.7 percentage points; 95% CI 28.4 to 32.8), followed by Black race (+16.3 percentage points; 95% CI 14.3 to 18.4) and Asian race (+12.8 percentage points; 95% CI 11.0 to 14.6). High-status occupation was associated with a 19.5-percentage-point reduction (95% CI −21.8 to −17.3), friendship with institutional leadership with a 19.4-point reduction (95% CI −21.5 to −17.2), major donor status with an 18.0-point reduction (95% CI −20.2 to −15.8), and a large public following with a 16.1-point reduction (95% CI −18.1 to −14.1). Across all individual attribute contrasts, position-balanced predicted selection probabilities ranged from 30.5% for high-status occupation versus hourly/service work to 80.7% for Indigenous versus White race, a 50.2-percentage-point range. This range compares individual attribute contrasts and does not represent a comparison between combined multi-attribute patient profiles.

### 3.4. Context-Dependency of Allocation Decisions

Context-specific A + B models demonstrated that the direction of several associations was consistent, although effect magnitudes varied by vignette (Supplementary Table S1). Black race was associated with higher odds of selection in all seven scenarios, with position-averaged ORs ranging from 5.82 (95% CI 4.25–7.95) in the expedited specialty visit vignette to 14.17 (95% CI 10.60–18.94) in the behavioral health fast-track vignette. Woman gender identity was also associated with higher odds of selection in all seven vignettes, ranging from OR 1.54 (95% CI 1.27–1.86) in high-intensity social work to OR 3.53 (95% CI 2.87–4.34) in foundation support. High-status occupation was associated with lower odds of selection in every vignette and was most pronounced in foundation support (OR 0.014, 95% CI 0.010–0.020; FDR-adjusted *p* < 0.001).

### 3.5. Internal Model Consistency and Stability

Quantitative priority scores showed a pattern broadly concordant with the binary allocation decisions (Table 3). In the position-averaged linear model, the largest positive score difference was associated with Indigenous versus White race (+16.89 points), followed by Black versus White race (+12.87 points) and Asian versus White race (+9.33 points). The largest negative differences were associated with high-status versus hourly/service occupation (−16.70 points), major donor versus no donor status (−16.34 points), and friendship with institutional leadership versus no connection (−13.18 points). All 17 position-averaged score contrasts remained statistically significant after false discovery rate correction.

The alignment between the model’s qualitative choices and its quantitative scoring was high (Supplementary Table S2). The applicant with the higher priority score was the chosen recipient in 95.1% of all trials. This concordance was high across all vignettes, ranging from 90.5% to 99.7%. Stability analysis of repeated identical prompts (n = 30 per vignette) showed excellent reproducibility, with an aggregate choice agreement rate of 92.9%. In four of the seven vignettes, the model’s decision was identical in 100% of trials, while the remaining three vignettes exhibited agreement rates ranging from 76.7% to 90.0%. The priority scores themselves showed moderate variance across repetitions (mean coefficient of variation = 0.210).

## 4. Discussion

In this systematic algorithmic audit, the gpt-5-chat-latest model alias produced substantial allocation differences across demographic and social descriptors despite identical stated clinical need, prognosis, and expected benefit. Race had the largest descriptive grouped association, followed by occupation, donor status, institutional connection, public visibility, age, gender identity, and presentation. The direction of effects was heterogeneous: Indigenous, Black, and Asian race and woman and non-binary gender identity were associated with higher selection odds, whereas high-status occupation, institutional connection, donor status, and public visibility were associated with lower odds. Across individual attribute contrasts, position-balanced predicted selection probability ranged from 30.5% for high-status occupation versus hourly/service work to 80.7% for Indigenous versus White race, a 50.2-percentage-point range. This comparison reflects single-attribute contrasts rather than combined multi-attribute patient profiles.

A central finding of this study is the model’s inversion of the preferential treatment often documented in human healthcare systems. In clinical sociology, this is frequently described as the accumulation of advantage, where patients with wealth, influence, or connections to hospital leadership secure expedited access to scarce resources [19]. The LLM system, conversely, exhibited a robust inverse association between status and allocation. Patients described as friends of leadership, major donors, or corporate executives received significantly lower allocation scores. These individuals were effectively deprioritized based on their social capital. The present design cannot determine whether this pattern arose from pretraining data, post-training alignment, prompt interpretation, or other components of the model pipeline. Regardless of mechanism, the findings demonstrate systematic use of clinically irrelevant information in the allocation task [1]. Medical ethics traditionally relies on the principle that allocation must be determined by prognosis, urgency, and capacity to benefit [20]. However, triage ethics are pluralistic and encompass a broad spectrum of valid moral frameworks. For instance, prioritarian or ‘worst-off’ frameworks argue that it is ethically defensible to favor the most vulnerable or historically marginalized groups to correct systemic inequities [16]. Alternatively, utilitarian models advocate for maximizing overall societal benefit, a calculus that some could controversially extend to socioeconomic considerations [21]. While our descriptive findings highlight a stark deviation from strict clinical neutrality, this deviation is not inherently malicious under all ethical lenses. Under a strict clinical-neutrality framework, such non-clinical weighting would be impermissible, whereas prioritarian frameworks may interpret some preferences for disadvantaged patients differently. The central governance concern is that these trade-offs are implicit rather than explicitly specified, reviewable, or accountable, because value-laden triage decisions based on unexplained weighting of social variables conflict with the norms of transparent clinical governance.

Age represented a separate and ethically important source of variation. Patients aged 90 years had substantially lower position-averaged odds of selection than otherwise equivalent 60-year-old patients (OR 0.23, 95% CI 0.22–0.25), corresponding to an 8.9-percentage-point reduction in position-balanced selection probability. In contrast, the difference between patients aged 30 and 60 years was not statistically significant for the binary allocation outcome. The role of age in scarce-resource allocation remains ethically contested. Life-cycle or “fair innings” approaches may permit younger patients to receive priority on the grounds that they have had less opportunity to experience the stages of life, whereas egalitarian frameworks generally reject chronological age alone as a basis for unequal treatment [22]. Other allocation frameworks permit age to influence prioritization indirectly when it is associated with prognosis, expected duration of benefit, or life-years saved [23]. Our experiment is distinct because clinical need, prognosis, and expected benefit were explicitly stipulated to be identical between the competing patients. The residual disadvantage associated with age 90 therefore cannot be attributed within the prompt to poorer clinical prognosis or reduced expected benefit. This finding illustrates that opaque non-clinical weighting in LLM allocation is not limited to socioeconomic status and warrants explicit evaluation of age-related behavior in future clinical AI audits.

Our analysis revealed a high degree of internal consistency between the model’s qualitative choices and quantitative scoring, with choice-score concordance of 95.1%. Together with the stability repeats, this suggests that the observed allocation patterns were not solely attributable to stochastic variability. Four of seven vignettes showed 100% agreement with the modal choice across repeated prompts, while agreement in the remaining three ranged from 76.7% to 90.0%. From a systems perspective, reproducible non-clinical weighting could be propagated at scale if embedded within decision-support workflows. Stable quantitative scores may therefore create an appearance of objectivity even when recommendations are influenced by characteristics unrelated to clinical need, prognosis, or expected benefit.

We also observed context-dependent variation in effect magnitude, although the direction of several associations remained consistent. Woman gender identity was associated with higher selection odds in all seven vignettes, but the association was weakest in high-intensity social work and strongest in foundation support. High-status occupation was associated with lower odds in every vignette, with the largest penalty in foundation support. This latter context may have encouraged the model to infer financial need from occupation despite the prompt’s explicit statement of equal clinical need, prognosis, and expected benefit. These findings underscore the need to audit LLMs across diverse task formulations because contextual wording can interact with non-clinical patient descriptors.

These findings have specific implications for the governance and design of clinical LLM systems. First, the opacity of these preferences is incompatible with the requirement for transparency in health policy [24]. In healthcare systems where distributive justice must be publicly defensible, allocation criteria must be explicit. If an LLM allocates a scarce resource to one patient over another, it cannot justify that decision based on the grooming standards or institutional connections of the patients. Relying on opaque algorithmic logic for such fundamental choices undermines the accountability required in modern clinical practice.

Second, our results show that a general-purpose aligned LLM can exhibit substantial non-clinical allocation differences even when clinical equivalence is explicitly stipulated. Because this study cannot identify whether these patterns originated in pretraining, post-training alignment, prompt interpretation, or other model components, they should not be attributed to alignment mechanisms specifically. Developers should therefore implement safeguards such as restricting allocation models to clinically relevant inputs or explicitly excluding non-medical descriptors when they have no legitimate role in the decision.

Third, the presentation of model-generated priority scores to clinicians carries a risk of automation bias. These scores varied across repeated prompts, with a mean coefficient of variation of 0.21, and were systematically associated with non-clinical patient characteristics. If clinicians interpret such scores as objective measures of medical urgency, they may inadvertently defer to a metric influenced by attributes unrelated to clinical need, prognosis, or expected benefit. Risk management guidelines for LLMs in safety-critical domains emphasize the need for traceable links between model outputs and clinical justifications [25]. Consequently, realistic clinical integration should preserve identifiable human responsibility for allocation decisions, with LLMs functioning as decision support tools rather than autonomous decision makers. This approach is consistent with recent accountability frameworks emphasizing clear responsibility attribution, institutional policies, and continued independent clinical judgment when AI systems inform patient care [26]. Implementing workflow safeguards, such as cognitive forcing functions that require clinicians to verify clinical rationales before viewing quantitative scores, could help mitigate automation bias. Deployment should also incorporate ongoing post-deployment monitoring and standardized local auditing for non-clinical allocation differences.

### Strengths and Limitations

This study utilized a full-factorial design that effectively disentangled collinear social variables. The large sample size allowed for the precise estimation of effect sizes, and the use of stateless prompts isolated the model’s behavior from the influence of conversational history. By quantifying both relative odds and absolute risk differences, we provided a detailed characterization of the algorithmic decision plane. Furthermore, the inclusion of stability repeats helped distinguish stochastic variability from reproducible response patterns.

However, several limitations apply. We evaluated the gpt-5-chat-latest API model alias at a single point in time, October 8, 2025; therefore, our findings should be interpreted as specific to the model behavior observed on that date rather than as a universal property of GPT-5-family models or LLMs more broadly. Given the frequency of model updates and differences across model architectures, these effect estimates are subject to change. Future research should evaluate these scenarios across multiple open-source and proprietary model families to determine whether these effects are model-specific or generalizable across architectures and training paradigms. The profiles were also textual and synthetic. They lacked the complexity and heterogeneity of real electronic health records, such as diagnostic uncertainty, missing data, and evolving clinical trajectories, which limits the ecological validity of the findings. The forced-choice design required a decision even in cases of clinical equipoise where randomization might be the ethical standard. By eliminating the option to defer to a human clinician, express uncertainty, or recommend randomization, this prompt structure creates conditions in which non-clinical descriptors or display-position effects can act as tiebreakers. This design choice likely amplifies the observed behavioral differences and may overestimate the real-world magnitude of these biases compared to a system that allows for clinical equipoise. Furthermore, these simulated environments lack the human oversight and multidisciplinary discussions characteristic of actual clinical triage. Consequently, these findings reflect the isolated baseline behavior of the algorithm rather than outcomes in a fully deployed healthcare system. Additionally, our statistical approach focused on estimating the main marginal effects of each attribute. While this establishes a baseline hierarchy of variable importance, it does not explore intersectional interaction effects, such as how the penalty for high occupational prestige might differ across racial or gender identities. Future research should utilize targeted intersectional designs to explore these complex subgroups. Finally, while we identified behavioral bias in the model’s outputs, our analysis does not elucidate the specific mechanisms within the training or alignment pipeline that produced these results.

## 5. Conclusions

As LLMs are increasingly considered for clinical applications, their allocation behavior should be evaluated not only for accuracy but also for invariance to patient characteristics that are irrelevant to the clinical decision [27]. In this controlled audit, a general-purpose LLM assigned substantially different priorities to clinically equivalent patients when demographic and social descriptors were changed. These findings reflect the behavior of a specific API model alias as queried on 8 October 2025 under synthetic forced-choice conditions and should not be interpreted as evidence that the same effects would necessarily occur in deployed clinical decision-support systems. They nevertheless demonstrate that a safety-aligned general-purpose model is not thereby neutral when asked to resolve clinical equipoise.

Mitigation should therefore operate at both the system-design and validation levels. Clinical implementations could restrict allocation models to structured, clinically relevant inputs; apply preprocessing or feature-gating layers that exclude normatively irrelevant descriptors such as donor status, public visibility, or institutional connections when these variables have no legitimate role in the decision; and require explicit clinical justification for any generated priority score or recommendation. Before deployment, counterfactual invariance testing should systematically vary protected and social attributes while holding clinical information constant, with predefined thresholds for unacceptable changes in recommendations. Because our analysis also identified substantial sensitivity to patient display position, future evaluations should randomize presentation order and test position-specific effects rather than assuming symmetric responses. Model versioning, auditable decision logs, repeated testing after model updates, and prospective monitoring for distributional shifts should complement continuous human oversight. Future work should determine whether these safeguards reduce non-clinical allocation disparities across multiple proprietary and open-source models without degrading clinically appropriate discrimination based on prognosis, urgency, or expected benefit.

## Figures and Tables

**Table 1:** Position-Averaged Associations Between Patient Characteristics and Resource Allocation.

Characteristic	Odds Ratio (OR)	95% Confidence Interval (CI)	FDR-Adjusted *p*-Value
Age			
30 years	1.062	0.990–1.139	0.092
60 years	1.000 (Reference)		
90 years	0.234	0.217–0.253	<0.001
Race			
White	1.000 (Reference)		
Black	8.073	7.321–8.902	<0.001
Asian	4.833	4.410–5.296	<0.001
Indigenous	16.477	14.854–18.277	<0.001
Gender Identity			
Man	1.000 (Reference)		
Woman	2.350	2.183–2.529	<0.001
Non-binary individual	3.716	3.440–4.013	<0.001
Presentation & Grooming			
Casual	1.000 (Reference)		
Professional	0.796	0.742–0.854	<0.001
Disheveled	1.933	1.798–2.077	<0.001
Occupational Prestige			
Hourly/Service	1.000 (Reference)		
Unemployed	0.679	0.633–0.729	<0.001
High status	0.064	0.058–0.071	<0.001
Institutional Connection			
None	1.000 (Reference)		
Knows someone	0.454	0.422–0.489	<0.001
Friends with leader	0.121	0.111–0.131	<0.001
Public Visibility			
Not publicly known	1.000 (Reference)		
Locally known	0.461	0.429–0.496	<0.001
Large following	0.149	0.137–0.161	<0.001
Donor Status			
None	1.000 (Reference)		
Small recurring	0.705	0.657–0.756	<0.001
Major donor	0.092	0.084–0.101	<0.001

Results are position-averaged odds ratios from the primary pooled logistic regression model, which included separate Patient A and Patient B attribute terms and vignette-specific position effects. An OR > 1 indicates higher odds of selection relative to the specified reference level, whereas an OR < 1 indicates lower odds. Benjamini–Hochberg false discovery rate correction was applied across the 17 primary attribute contrasts.

**Table 2:** Position-Balanced Risk Differences in Allocation Probability by Patient Characteristic.

Characteristic	Risk Difference (Percentage Points)	95% Confidence Interval (CI)
Age		
30 years	+0.4	−0.1 to +0.9
90 years	−8.9	−10.5 to −7.6
Gender Identity		
Woman	+6.8	+5.7 to +8.0
Non-binary individual	+10.0	+8.6 to +11.6
Race		
Black	+16.3	+14.3 to +18.4
Asian	+12.8	+11.0 to +14.6
Indigenous	+30.7	+28.4 to +32.8
Presentation & Grooming		
Professional	−1.7	−2.3 to −1.1
Disheveled	+3.8	+3.1 to +4.6
Occupational Prestige		
Unemployed	−2.6	−3.3 to −2.0
High status	−19.5	−21.8 to −17.3
Institutional Connection		
Knows someone	−6.8	−8.0 to −5.7
Friends with leader	−19.4	−21.5 to −17.2
Public Visibility		
Locally known	−5.8	−6.9 to −4.8
Large following	−16.1	−18.1 to −14.1
Donor Status		
Small recurring	−2.8	−3.5 to −2.1
Major donor	−18.0	−20.2 to −15.8

Risk differences are expressed in percentage points and were derived from position-balanced predicted selection probabilities from the primary model. For each contrast, the focal attribute was averaged across Patient A and Patient B display positions within each vignette and then averaged equally across the seven vignettes. The reference-versus-reference selection probability was 50%. Ninety-five percent confidence intervals were obtained from 5000 draws from the multivariate normal distribution defined by the fitted coefficient vector and covariance matrix.

**Table 3:** Position-Averaged Differences in Allocation Priority Scores by Patient Characteristic.

Characteristic	Beta Coefficient (β)	95% Confidence Interval (CI)	FDR-Adjusted *p*-Value
Age			
30 years	1.30	0.83 to 1.77	<0.001
60 years	0.00 (Reference)		
90 years	−5.08	−5.55 to −4.61	<0.001
Gender Identity			
Man	0.00 (Reference)		
Woman	6.09	5.62 to 6.57	<0.001
Non-binary individual	8.24	7.76 to 8.71	<0.001
Race			
White	0.00 (Reference)		
Black	12.87	12.32 to 13.41	<0.001
Asian	9.33	8.79 to 9.88	<0.001
Indigenous	16.89	16.34 to 17.44	<0.001
Presentation & Grooming			
Casual	0.00 (Reference)		
Professional	−2.02	−2.49 to −1.54	<0.001
Disheveled	5.40	4.93 to 5.87	<0.001
Occupational Prestige			
Hourly/Service	0.00 (Reference)		
Unemployed	−1.71	−2.18 to −1.23	<0.001
High status	−16.70	−17.17 to −16.23	<0.001
Institutional Connection			
None	0.00 (Reference)		
Knows someone	−5.35	−5.82 to −4.88	<0.001
Friends with leader	−13.18	−13.65 to −12.71	<0.001
Public Visibility			
Not publicly known	0.00 (Reference)		
Locally known	−4.41	−4.88 to −3.94	<0.001
Large following	−10.49	−10.97 to −10.02	<0.001
Donor Status			
None	0.00 (Reference)		
Small recurring	−3.32	−3.79 to −2.85	<0.001
Major donor	−16.34	−16.81 to −15.87	<0.001

Results are from a pooled linear regression of the difference in priority scores (ScoreA minus ScoreB) containing separate Patient A and Patient B attribute terms and vignette fixed effects. For each characteristic, the reported coefficient is the position-averaged score effect, calculated as (βA − βB)/2, and summarizes the estimated change in priority score across both display positions relative to the specified reference level. Positive values indicate higher priority scores and negative values indicate lower priority scores. Benjamini–Hochberg false discovery rate correction was applied across the 17 score contrasts.

## Data Availability

The datasets used and/or analysed during the current study are available from the corresponding author upon reasonable request.

## Supplementary Materials

The following supporting information can be downloaded at https://www.mdpi.com/article/10.3390/jpm16090448/s1. Supplementary Methods S1. The Vignettes; Supplementary Methods S2. Schema for Demographic and Status Attribute Rendering; Supplementary Methods S3. Prompt Template and System Instructions; Supplementary Table S1. Context-Specific Position-Averaged Associations With Allocation Across Seven Vignettes; Supplementary Table S2. Choice-Score Alignment Metrics by Vignette; Supplementary Table S3. Sensitivity Analysis of Individual High-Status Occupational Exemplars; Supplementary Table S4. Position-Specific Attribute Coefficients and Tests of A/B Effect Symmetry in the Primary Forced-Choice Model.
